# Evaluating sequence contributions to MRI radiomics for glioblastoma survival: single vs fusion models

**DOI:** 10.3389/fonc.2025.1743451

**Published:** 2026-01-12

**Authors:** Ruirui Guo, Ya Gao

**Affiliations:** 1 Department of Radiology, Shenmu Hospital, Shenmu, Shaanxi, China; 2 Department of Functional Sciences, Shenmu Hospital, Shenmu, Shaanxi, China

**Keywords:** classification comparison, quantitative imaging features, radiomic, structural MRI, survival

## Abstract

**Background:**

Glioblastoma is the most aggressive primary brain tumor with a poor prognosis. Multiparametric MRI-based radiomics shows promise for prognosis, yet most studies fuse all sequences without quantifying their individual contributions. We systematically compared the prognostic value of features from single sequences and comprehensive fusions for survival classification.

**Methods:**

This retrospective study included glioblastoma patients from TCIA. Quantitative features were extracted from T1, T1-GD, T2, and T2-FLAIR images. Univariate ROC analyses with Benjamini-Hochberg false discovery rate correction assessed each feature’s prognostic value. For multivariate modeling, combining univariate analysis with the maximum relevance minimum redundancy algorithm, was used to select the top predictors for each model. Logistic regression models were then built using features from single sequences, dual-sequence fusions, and a comprehensive four-sequence fusion. Performance was compared using repeated random subsampling validation with AUC as the metric.

**Results:**

Univariate analysis identified 249 features with significant prognostic power (T1-GD: 79; T2: 58; T2-FLAIR: 57; T1: 55). In the multivariate analysis, the four-sequence fusion model achieved the highest performance on the training cohort (AUC: 0.8467), but this advantage did not generalize to the validation cohorts. On the validation set, single-sequence models achieved AUCs ranging from 0.6599 to 0.7010, with the T2 model performing best. The dual-sequence model combining features from T1-GD and T2 sequences yielded the highest overall performance, achieving a mean validation AUC of 0.7066. Notably, this targeted two-sequence model outperformed the more complex four-sequence model (AUC: 0.7030).

**Conclusions:**

For glioblastoma survival prediction, a strategic selection of complementary imaging sequences might be more effective than an indiscriminate aggregation of all available structural MRI data. However, this finding is currently specific to this particular dataset and clinical task. Future research using large-scale data with multiple tumor types and clinical objectives is necessary to explore the broader generalizability of these results.

## Introduction

Glioblastoma is the most common and aggressive primary brain tumor in adults, with a prognosis that remains exceptionally poor despite multimodal treatment (1, 2). The median survival time has seen little improvement over the past decade, highlighting an urgent need for reliable prognostic biomarkers (3, 4). Such biomarkers are critical for patient counseling, clinical trial stratification, and guiding personalized therapeutic strategies (5). While molecular markers are valuable, they require invasive tissue sampling (6). This limitation has fueled interest in non-invasive methods, positioning medical imaging at the forefront of prognostic research.

Medical imaging, particularly multiparametric Magnetic Resonance Imaging (MRI), has emerged as a cornerstone in the management of glioblastoma, providing critical information on tumor location, size, and relationship with surrounding structures (7). The emerging field of radiomics, which extracts high-throughput quantitative features from these images, shows significant promise for developing powerful prediction models (8–13). The standard glioblastoma protocol includes several MRI sequences, each offering unique biological insights. For instance, post-contrast T1-weighted (T1-Gd) images delineate the active tumor core, while T2-weighted (T2) and T2 Fluid-Attenuated Inversion Recovery (T2-FLAIR) sequences excel at visualizing surrounding edema and non-enhancing tumor (14, 15).

Given this multiparametric approach, most radiomic studies operate on a prevailing assumption: that fusing features from all available sequences will yield the most powerful and comprehensive model (16, 17). However, this “more is better” strategy has a critical, often unexamined, drawback. Indiscriminately aggregating data can introduce information redundancy and noise, increasing model complexity without a proportional gain in prognostic value. More importantly, this complexity raises the risk of overfitting, where a model performs exceptionally well on training data but fails to generalize to new, unseen cases. To date, few studies have systematically dismantled this fusion-based approach to quantify the actual prognostic contribution of each individual sequence.

Despite the rapid growth of GBM radiomics using multiparametric MRI, the optimal scope of sequence inclusion remains uncertain. Many studies report improved performance with multi sequence inputs. However, fewer works have performed a controlled comparison of reduced sequence sets versus full structural protocols for survival related endpoints. Such comparisons are often confounded by differences in classifiers, feature selection strategies, or feature counts. As a result, it remains unclear whether broader fusion truly adds task specific value beyond a targeted subset of sequences.

Therefore, this study challenges the conventional fusion paradigm. Our primary objective was to systematically evaluate the independent and combined prognostic value of features from four standard MRI sequences for glioblastoma survival classification. We hypothesized that a strategic combination of complementary sequences would produce a more robust and generalizable model than an indiscriminate, all-inclusive fusion. To test this, we constructed and rigorously compared the performance of models built from single sequences, dual-sequence fusions, and a comprehensive four-sequence fusion. This work seeks to clarify the contribution of each MRI sequence, providing crucial insights for designing more efficient and effective radiomics models in neuro-oncology.

## Materials and methods

### Study design and patient dataset

**Figure 1:**
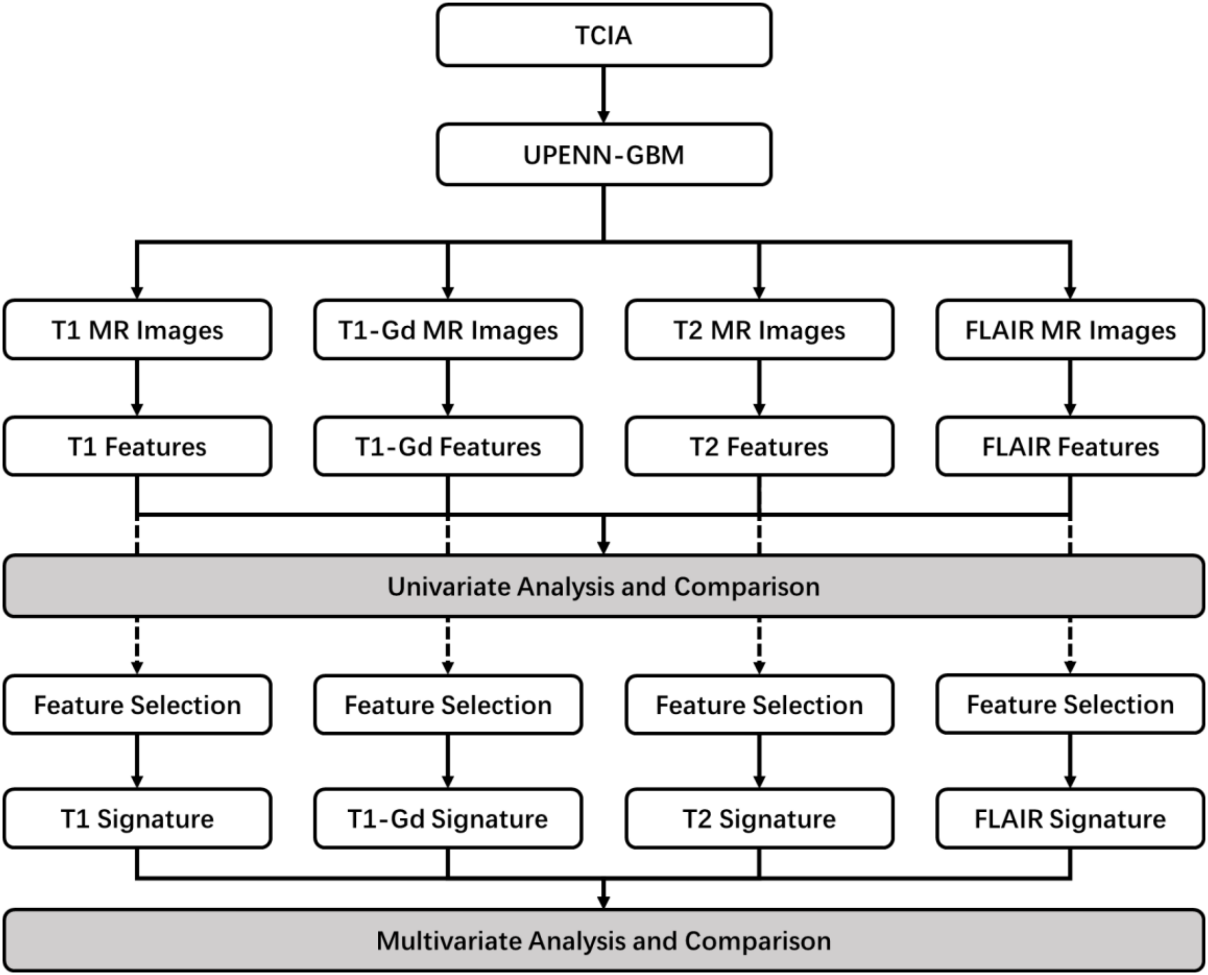
The workflow of this study. The workflow begins with data acquisition from the TCIA database. Subsequent stages include a univariate analysis for initial feature screening, and a multivariate analysis involving feature selection and the construction of single-sequence and multi-sequence prognostic models for comparison.

Our study workflow consisted of two main stages: univariate and multivariate analysis (**Figure 1**). We obtained a retrospective dataset of 227 glioblastoma patients from The Cancer Imaging Archive (TCIA) (18, 19). For each patient, we used multiparametric MRI data, including T1-weighted (T1), post-contrast T1-weighted (T1-Gd), T2-weighted (T2), and T2 fluid-attenuated inversion recovery (T2-FLAIR) sequences. All images underwent a uniform preprocessing pipeline before feature extraction. After de identification and de facing, scans were maintained in their original resolution and orientation. Brain extraction and tumor sub region segmentation were then applied. The regions of interest included the necrotic tumor core, the enhancing tumor, and the peritumoral edematous or infiltrated tissue. The enhancing tumor and necrotic core were identified based on contrast related intensity differences on T1 Gd relative to T1 and in reference to normal appearing white matter. The necrotic core encompassed non enhancing or faintly enhancing tumor components along with transitional and necrotic regions that are commonly resected together with the enhancing component. The edema region was delineated by the hyperintense signal envelope on T2 FLAIR images.

We defined the survival endpoint as one-year overall survival and performed binary classification based on whether patients were alive at 12 months after diagnosis. The demographic details of these patients were summarized in **Table 1**.

**Table 1:** Demographics of the dataset for this study.

Demographics	Value	Number	Percentage
Gender	Male	94	41.41%
	Female	133	58.59%
Age (years)	≤30	2	0.88%
	30-50	28	12.33%
	51-70	132	58.15%
	>70	65	28.63%
Resection Status	Gross Total	156	68.72%
	Partial	63	27.75%
	Not Available	8	3.52%
Survival Status (years)	≤1	102	44.93%
	>1	125	55.07%

Tumor segmentations and the corresponding pre computed radiomic feature tables were obtained from the previously published resource associated with the UPenn GBM collection on TCIA. The original resource provided standardized tumor subregion masks for the cohort. We used these segmentations as provided. We did not perform additional manual editing or re segmentation. The radiomic features for each MRI sequence were computed in the original work using a consistent extraction configuration. We adopted the same feature tables for all single sequence and fusion analyses. This design ensures that our comparisons across sequences were based on a shared upstream segmentation and feature extraction framework.

To enhance reproducibility, we utilized pre-calculated radiomic features from a prior study. These features were originally extracted using the PyRadiomics library in compliance with the Image Biomarker Standardisation Initiative (IBSI) guidelines. A total of 432 quantitative features were available for each MRI sequence. Detailed descriptions of the image preprocessing and feature extraction are available in the previous studies (12, 18).

### Univariate analysis

First, we assessed the prognostic value of each individual radiomic feature using univariate receiver operating characteristic (ROC) analysis (20). The area under the ROC curve (AUC) served as the primary metric to quantify discriminatory performance, where an AUC of 1.0 represents a perfect classifier and 0.5 indicates performance equivalent to random chance. We determined the statistical significance of each feature by testing its AUC against a null value of 0.5 with the DeLong test (21).

To account for multiple comparisons arising from the large feature set, the raw p-values were adjusted using the Benjamini-Hochberg procedure to control the false discovery rate (FDR) (22). A feature was considered to possess statistically significant discriminatory power if its corresponding FDR-adjusted P-value was less than 0.05.

### Multivariate analysis

To build robust models, we implemented a repeated random sub-sampling validation strategy. We repeated the entire model development and evaluation process 10 times. In each iteration, we randomly partitioned the patients into a training cohort and a validation cohort at a ratio of 3:1. All feature values were normalized prior to feature selection.

Our multivariate analysis employed a two-stage feature selection process (23, 24). First, we screened features based on the univariate analysis, retaining only those with significant prognostic value (FDR-adjusted p < 0.05). This step reduced feature space dimensionality and filtered out potential noise. Second, we applied the maximum relevance minimum redundancy (mRMR) algorithm to this reduced set. The mRMR algorithm selects an optimal feature subset by maximizing relevance to the outcome while minimizing redundancy among features (25). The mRMR feature selection approach was implemented in R software using the “mRMRe” package (26). Univariate screening and mRMR were performed independently within each repeated split using only the training subset, and the selected features were used to train the classifier before evaluation on the held-out validation subset.

To ensure a fair and standardized comparison between models, we fixed the feature subset size at 10 for all models. We then used a logistic regression algorithm to construct the classifiers. This consistent framework allowed for an objective evaluation of three model categories:

(1) single-sequence models, which were constructed using significant features derived exclusively from one of the four sequences (i.e., T1-only, T1-GD-only, T2-only, and T2-FLAIR-only); (2) dual-sequence fusion models, which combined significant features from pairs of complementary sequences; and (3) a comprehensive multi-sequence fusion model, which incorporated significant features from all four sequences simultaneously.

It was pertinent to clarify that the primary goal of this study was not to build the single best prognostic model, but rather to systematically compare the predictive performance of these different MRI feature sets. Our methodology was therefore designed to support this specific comparative objective.

## Results

### Univariate analysis and comparison

From an initial set of 1728 radiomic features, with 432 extracted from each of the four MRI sequences (T1, T1-GD, T2, and T2-FLAIR), the univariate ROC analysis was performed to identify individual predictors with significant prognostic value. After statistical analysis using the DeLong test and subsequent correction for multiple comparisons with the Benjamini-Hochberg procedure, 249 of the 1728 features were identified as having statistically significant discriminatory power (all FDR-adjusted P-values < 0.05).

**Figure 2:**
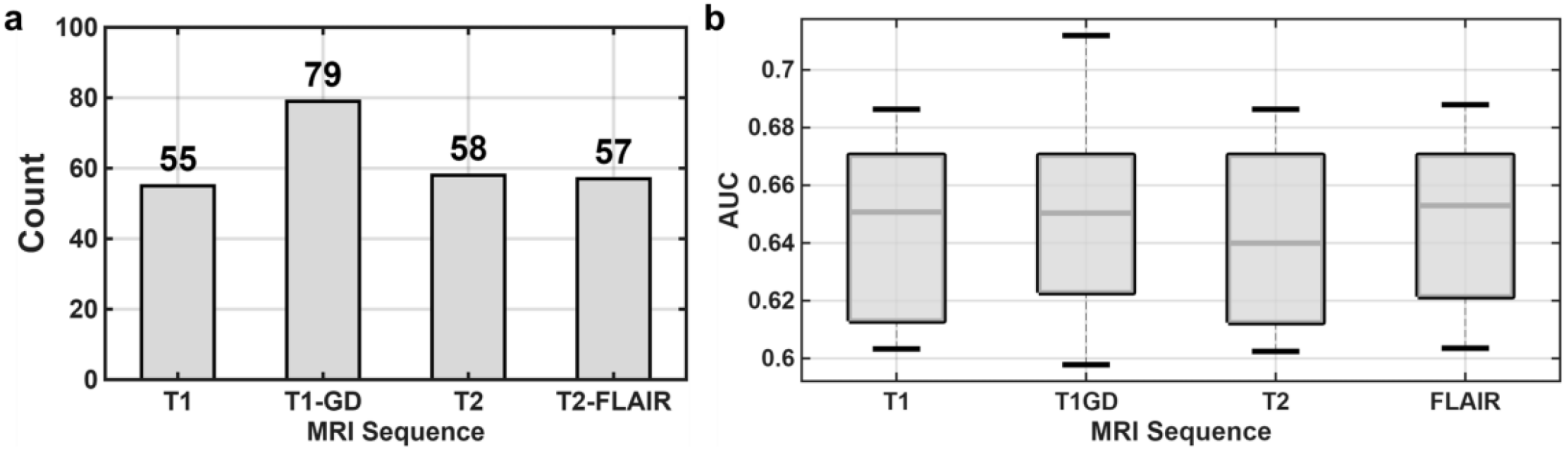
Univariate analysis of significant prognostic features from different MRI sequences. **(a)** Bar chart illustrating the number of statistically significant features identified from each of the four MRI sequences (T1, T1-GD, T2, and T2-FLAIR). **(b)** Boxplot comparing the distribution of the AUC values for the significant features from each sequence.

The prognostic performance and distribution of these 249 significant features across the different sequences are summarized in **Table 2**. Specifically, the T1-GD sequence yielded the highest number of significant predictors (n=79), followed by T2 (n=58), T2-FLAIR (n=57), and T1 (n=55) (**Figure 1a**). The retained features from all sequences demonstrated modest but consistently significant predictive capabilities. The mean AUC values were comparable across the modalities, with features from the T2-FLAIR sequence exhibiting the highest average performance (Mean AUC = 0.6483 ± 0.0266; Median AUC = 0.6530) (**Figure 2b**). Notably, the single feature with the highest individual prognostic value originated from the T1-GD sequence, achieving a maximum AUC of 0.7119. Overall, the AUC values for all significant features ranged from a minimum of 0.5978 to a maximum of 0.7119.

**Table 2:** Univariate analysis for individual imaging feature.

Term	T1	T1-GD	T2	T2-FLAIR
Number	55	79	58	57
AUC: Mean ± STD	0.6447 ± 0.0285	0.6470 ± 0.0280	0.6410 ± 0.0279	0.6483 ± 0.0266
AUC: Median	0.6507	0.6504	0.6400	0.6530
AUC: Range	0.6033, 0.6864	0.5978, 0.7119	0.6024, 0.6864	0.6035, 0.6879

### Multivariate analysis and comparison

**Figure 3:**
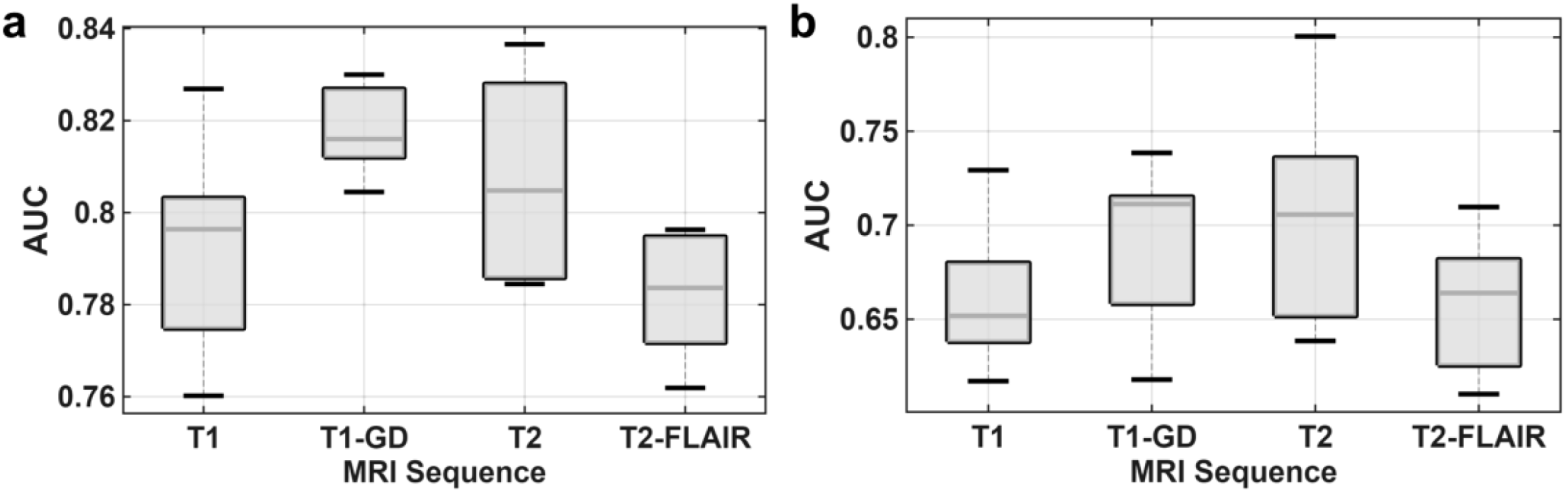
Performance of single-sequence prognostic models. Boxplots comparing the distribution of AUC values for models constructed from each of the four individual MRI sequences across 10 independent experiments. **(a)** Performance on the training cohorts. **(b)** Performance on the corresponding validation cohorts.

In line with our initial hypothesis, the multivariate analysis revealed distinct prognostic capabilities among the models derived from different MRI sequences. Following the feature selection protocol, we constructed and evaluated three categories of multivariate models: single-sequence, dual-sequence fusion, and a comprehensive four-sequence model. The performance of the single-sequence models is visually summarized in **Figure 3** and detailed in **Table 3**. For the single-sequence models, the mean AUC values on the validation cohorts ranged from 0.6599 to 0.7010 (**Figure 3b**). Among the four individual MRI sequences, the model derived from T2-weighted images demonstrated the highest prognostic performance, achieving a mean validation AUC of 0.7010. The performance of the T1-GD model was highly comparable, with a mean AUC of 0.6939, and there was no significant statistical difference between the T2 and T1-GD based models (P-value = 0.8789). In contrast, the T1 and T2-FLAIR models yielded slightly lower performances, with mean AUCs of 0.6605 and 0.6599, respectively. Although the T2-based model showed a trend towards better performance compared to the T1 and T2-FLAIR models, these differences did not reach statistical significance (P-value = 0.0753 and P-value = 0.0892, respectively).

**Table 3:** Detailed prognosis performance of single-sequence images using multivariate analysis.

Experiment No.	T1	T1-GD	T2	T2-FLAIR
	Training cohort			
1	0.8269	0.8277	0.8366	0.7963
2	0.7748	0.8096	0.7974	0.7619
3	0.7784	0.8203	0.7858	0.7800
4	0.8032	0.8300	0.828	0.7957
5	0.7923	0.812	0.8014	0.7717
6	0.7602	0.8126	0.8164	0.7681
7	0.7735	0.8045	0.8295	0.793
8	0.8043	0.8133	0.7845	0.7873
9	0.8020	0.8269	0.7853	0.7761
10	0.8005	0.8187	0.8082	0.7948
Mean	0.7916 ± 0.0197	0.8176 ± 0.0086	0.8073 ± 0.0196	0.7825 ± 0.0127
	Validation cohort			
1	0.6513	0.6179	0.6385	0.6556
2	0.6380	0.6582	0.7273	0.7020
3	0.6757	0.7275	0.8005	0.6740
4	0.6429	0.6393	0.6517	0.6102
5	0.6800	0.7071	0.7360	0.7097
6	0.6853	0.7121	0.7026	0.6819
7	0.6524	0.7152	0.6402	0.6254
8	0.6333	0.7385	0.7394	0.6723
9	0.6171	0.7105	0.7088	0.6248
10	0.7293	0.7121	0.6647	0.6431
Mean	0.6605 ± 0.0326	0.6939 ± 0.0404	0.7010 ± 0.0523	0.6599 ± 0.0338

**Figure 4:**
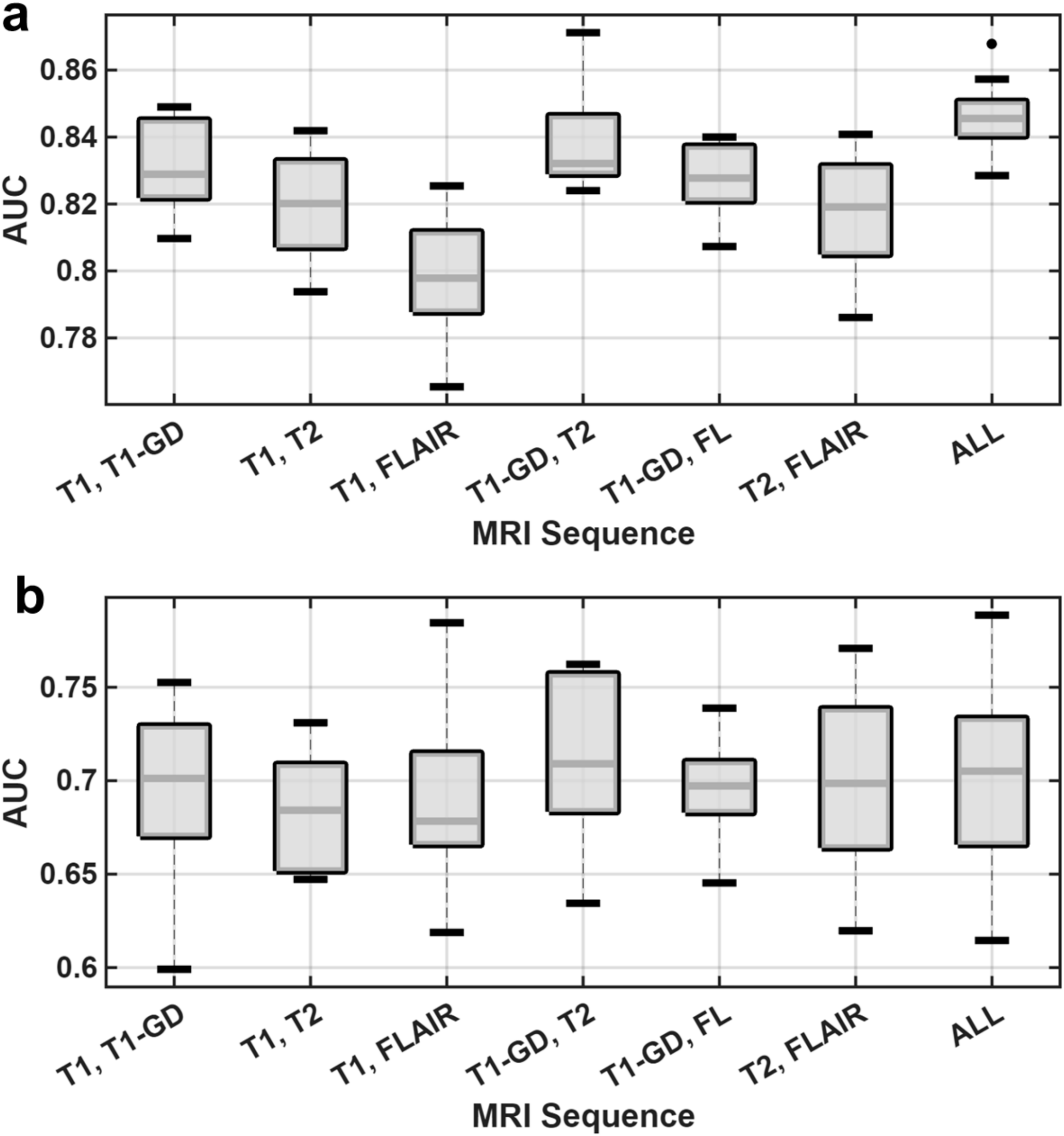
Performance comparison of dual-sequence and multi-sequence fusion models. Boxplots comparing the AUC distributions for models built using features from pairs of sequences and a comprehensive model using features from all four sequences (“ALL”). **(a)** Performance on the training cohorts. **(b)** Performance on the validation cohorts.

Next, we assessed the performance of the fusion models that combined features from multiple sequences. The results are visualized in **Figure 4** and summarized in detail in **Table 4**. On the training cohorts, fusing features consistently provided more effective information and yielded higher predictive performance (**Figure 4a**). For example, the comprehensive four-sequence model achieved the highest training AUC of 0.8467, and the best dual-sequence model (T1-GD + T2) reached a training AUC of 0.8384, both of which were higher than the best single-sequence model’s training performance (T1-GD, AUC = 0.8176). However, this performance enhancement was less consistent on the validation cohorts (**Figure 4b**). The dual-sequence model combining T1-GD and T2 features yielded the highest prognostic performance among all constructed models, with a mean validation AUC of 0.7066. The comprehensive model that integrated all four sequences also showed robust performance with a mean AUC of 0.7030. Despite the success of these top-performing fusion models, not all combinations resulted in improved accuracy. Several dual-sequence models, such as the one combining T1 and T2 features (mean AUC = 0.6836), resulted in lower validation performance than the best single-sequence models. These findings suggest that while feature fusion can be beneficial, the specific combination of T1-GD and T2 sequences provides the most synergistic and effective prognostic information for the validation set.

**Table 4:** Detailed prognosis performance of dual-sequence images using multivariate analysis.

No.	T1, T1-GD	T1, T2	T1, FLAIR	T1-GD, T2	T1-GD, FLAIR	T2, FLAIR	ALL
	Training cohort						
1	0.8480	0.8376	0.8254	0.8712	0.8378	0.8408	0.8573
2	0.8284	0.8070	0.7877	0.8240	0.8220	0.8101	0.8403
3	0.8235	0.7938	0.7965	0.8305	0.8289	0.8049	0.8381
4	0.8294	0.8329	0.8108	0.8313	0.8373	0.8291	0.8435
5	0.8218	0.8164	0.797	0.8274	0.8266	0.8015	0.8286
6	0.8097	0.8239	0.7654	0.8529	0.8073	0.8189	0.8492
7	0.8208	0.8419	0.7987	0.8329	0.8209	0.8314	0.8457
8	0.849	0.8148	0.8117	0.8289	0.8341	0.8193	0.8454
9	0.8451	0.802	0.773	0.8381	0.8185	0.7861	0.8507
10	0.8317	0.8306	0.8192	0.8464	0.8400	0.8366	0.8678
Mean	0.8307± 0.0130	0.8201± 0.0160	0.7985± 0.0193	0.8384± 0.0146	0.8273± 0.0104	0.8179± 0.0173	0.8467± 0.0107
	Validation cohort						
1	0.5991	0.6573	0.6872	0.6344	0.6453	0.6197	0.6145
2	0.6380	0.6650	0.6751	0.6380	0.6751	0.7475	0.6658
3	0.7521	0.7054	0.7148	0.7572	0.7301	0.7708	0.7886
4	0.6702	0.6517	0.6658	0.6834	0.6949	0.664	0.6958
5	0.708	0.7088	0.7182	0.7623	0.6995	0.7385	0.7453
6	0.7526	0.7310	0.7845	0.7112	0.7388	0.7095	0.7086
7	0.7064	0.6472	0.6577	0.6925	0.6829	0.6603	0.7195
8	0.6961	0.7148	0.6808	0.7581	0.6876	0.7224	0.7334
9	0.6842	0.6511	0.6188	0.7224	0.7097	0.6876	0.7020
10	0.7293	0.7034	0.6759	0.7069	0.7103	0.6741	0.6560
Mean	0.6936± 0.0485	0.6836± 0.0319	0.6879± 0.0441	0.7066± 0.0462	0.6974± 0.0271	0.6994± 0.0465	0.7030± 0.0492

### Feature comparison of the multi-sequence models

To investigate the contribution and stability of individual features in the multivariate models, we analyzed the selection frequency of each feature by the mRMR algorithm across the 10 independent iterations. The most frequently selected features from each of the four MRI sequences are detailed in **Tables 5**, **6**. The analysis revealed that certain features served as highly stable and robust predictors. From the T1-GD sequence, the feature *ET_Histogram_Bins-16_Bins-16_Bin-13_Probability* was exceptionally dominant, being selected in 9 out of the 10 iterations. Similarly, for the T2-FLAIR sequence, *ET_GLCM_Bins-16_Radius-1_Correlation* was a highly stable predictor, selected in 8 of the 10 iterations. For the T1 sequence, the most frequently selected feature was *NC_Histogram_Bins-16_Bins-16_Bin-9_Probability*, selected 6 times. In the T2 sequence, two features, *ED_Histogram_Bins-16_Bins-16_TenthPercentile* and *ED_NGTDM_Complexity*, shared the highest selection frequency, each chosen 4 times. Regarding the diversity of selected features, the T1-GD and T2 sequences each had 12 unique features that were selected at least once during the modeling process. In contrast, the T2-FLAIR sequence provided the most concentrated set of predictors, with only 6 unique features being selected in total across all iterations.

**Table 5:** The selected T1 and TA-GD features for the construction of multi-sequence models.

Sequence	Feature name	Count
T1	NC_Histogram_Bins-16_Bins-16_Bin-9_Probability	6
	NC_Morphologic_Elongation	5
	NC_GLRLM_Bins-16_Radius-1_ShortRunEmphasis	5
	ET_GLSZM_Bins-16_Radius-1_LowGreyLevelEmphasis	4
	ET_Morphologic_EllipseDiameter_Axis-2	3
	ET_GLRLM_Bins-16_Radius-1_ShortRunEmphasis	3
	ED_Intensity_Maximum	2
	ET_Intensity_Energy	1
	ET_Histogram_Bins-16_Bins-16_Bin-13_Frequency	1
	ED_Histogram_Bins-16_Bins-16_NinetyFifthPercentileMean	1
T1-GD	ET_Histogram_Bins-16_Bins-16_Bin-13_Probability	9
	ET_Intensity_Range	5
	NC_GLRLM_Bins-16_Radius-1_ShortRunEmphasis	3
	ET_Intensity_Maximum	3
	ET_GLSZM_Bins-16_Radius-1_GreyLevelNonUniformityNormalized	3
	ET_Histogram_Bins-16_Bins-16_Bin-3_Frequency	2
	NC_Morphologic_Elongation	1
	NC_GLRLM_Bins-16_Radius-1_LongRunEmphasis	1
	ET_Histogram_Bins-16_Bins-16_Bin-12_Probability	1
	ET_Morphologic_EllipseDiameter_Axis-2	1
	ET_GLCM_Bins-16_Radius-1_Correlation	1
	ET_GLSZM_Bins-16_Radius-1_ZoneSizeMean	1

**Table 6:** The selected T2 and T2-FLAIR features for the construction of multi-sequence models.

Sequence	Feature name	Count
T2	ED_Histogram_Bins-16_Bins-16_TenthPercentile	4
	ED_NGTDM_Complexity	4
	NC_GLRLM_Bins-16_Radius-1_LongRunHighGreyLevelEmphasis	3
	NC_GLRLM_Bins-16_Radius-1_ShortRunEmphasis	3
	NC_Intensity_Range	1
	NC_Morphologic_Elongation	1
	ET_Histogram_Bins-16_Bins-16_Bin-15_Probability	1
	ET_GLSZM_Bins-16_Radius-1_LargeZoneLowGreyLevelEmphasis	1
	ET_GLSZM_Bins-16_Radius-1_ZonePercentage	1
	ED_Histogram_Bins-16_Bins-16_CoefficientOfVariation	1
	ED_Morphologic_Roundness	1
	ED_GLRLM_Bins-16_Radius-1_LongRunHighGreyLevelEmphasis	1
T2-FLAIR	ET_GLCM_Bins-16_Radius-1_Correlation	8
	NC_Morphologic_Elongation	3
	ET_Intensity_Minimum	2
	NC_GLRLM_Bins-16_Radius-1_LongRunEmphasis	1
	ET_Intensity_NinetiethPercentile	1
	ED_Morphologic_Roundness	1

## Discussion

In this study, we conducted a systematic comparison of quantitative radiomic features derived from four structural MRI sequences to evaluate their independent and combined prognostic value for survival classification in glioblastoma. Our analysis yielded two principal findings. First, among the models built from individual sequences, the T2-weighted sequence demonstrated the highest prognostic performance. Second, and most importantly, our results revealed a notable divergence in model performance between the training and validation cohorts. While aggregating data from all four sequences achieved the highest performance on the training data, this advantage did not generalize to the validation set. In the validation cohorts, the dual-sequence model that strategically combined features from T1-Gd and T2 sequences yielded the highest prognostic accuracy among all constructed models, even outperforming the more complex four-sequence model.

The strong performance of the T1-Gd and T2 dual-sequence model may suggest a potential synergistic relationship between the biological information these sequences capture. Glioblastoma has two key pathological hallmarks. One is a highly proliferative core with a compromised blood-brain barrier. The other is an aggressively infiltrative margin extending into the brain parenchyma (1). The T1-Gd sequence is effective at delineating the enhancing tumor core, which reflects active neovascularity. In contrast, the T2-weighted sequence is highly sensitive to edema and non-enhancing tumor infiltration (7). Therefore, combining T1-Gd and T2 could provide a more complete radiomic signature. This signature may capture both the tumor’s aggressive core and its invasive periphery, which are critical factors for patient outcome.

Our finding that the four-sequence model was not optimal in the validation set highlights a crucial concept in radiomics. Adding more data does not always lead to better results due to the risk of information redundancy. For instance, sequences like T2 and T2-FLAIR both assess edema and may offer overlapping information. This could introduce noise and increase model complexity without adding significant prognostic value. This observation is consistent with other studies. These studies have also reported that models from a limited set of sequences can perform as well as, or even better than, those using a full multiparametric protocol (27). This emphasizes that strategic sequence selection may be more critical than simply aggregating all available data.

Our findings extend the existing GBM radiomics literature by focusing on sequence contribution under a controlled analytic framework. Prior studies have frequently adopted multi sequence protocols. Yet reduced set versus full protocol comparisons for survival stratification have not always been evaluated systematically within the same modeling constraints. In this study, we fixed the classifier and the number of selected features across settings. We also applied repeated splits. This design reduces methodological confounding and allows a clearer assessment of the incremental information provided by each sequence and fusion strategy. The observation that T1 Gd plus T2 can match or exceed four sequence fusion therefore supports task specific and purpose-oriented sequence selection rather than default comprehensive fusion.

A key strength of this study is its systematic and rigorous comparative framework. Many radiomics studies adopt a fusion-based approach, often assuming that combining all available imaging data will yield the best results. Our work challenges this assumption. Instead of only presenting a final fused model, we meticulously dissected the prognostic contribution of each individual MRI sequence. This head-to-head comparison provides a more granular understanding of which sequences offer the most valuable or potentially redundant information. Methodologically, the robustness of our findings is enhanced by several factors. First, the use of a publicly available dataset ensures transparency and allows for greater reproducibility of our results. Second, we implemented a repeated random sampling validation strategy, iterating the entire modeling process 10 times to ensure that our performance estimates are stable and not due to a single favorable data split. Finally, by fixing the feature subset size to 10 for all multivariate models, we established an equitable framework that allows for a fair and direct comparison of the predictive power inherent to different MRI feature sets. Together, these strengths ensure that our conclusions are based on a robust and unbiased analytical foundation.

By the feature comparison of the multi-sequence models, the most stable feature was ET_Histogram_Bins-16_Bins-16_Bin-13_Probability, which was selected in 9 of 10 splits. As a first order histogram descriptor within the enhancing tumor, this feature may capture the distribution of enhancement intensities. This may reflect vascular permeability and necrotic or viable tumor admixture. These processes are closely linked to more aggressive tumor behavior. In addition, the most stable T2 FLAIR feature was ET_GLCM_Bins-16_Radius-1_Correlation, selected in 8 of 10 splits. GLCM correlation is a texture measure that reflects local spatial dependency of signal. In this context, it may serve as a proxy for microstructural heterogeneity within or adjacent to the enhancing component as depicted on FLAIR. Nevertheless, we would like to emphasize that these interpretations remain theoretical and hypothesis driven in the context of the present study. We did not perform direct biological validation or imaging pathology correlation. Although prior studies have begun to explore associations between radiomic signatures and underlying tumor biology, such evidence remains limited in GBM and is still insufficient to establish robust, generalizable mechanistic explanations (28, 29). We also outline that future work will prioritize biological correlation studies, ideally integrating radiomics with molecular markers and pathology to validate and refine the proposed interpretations.

Several of the features that were repeatedly selected across splits belonged to first order and histogram-based categories. These features summarize the global distribution of voxel intensities within the tumor related regions. Their stable selection suggests that coarse intensity patterns may carry consistent prognostic signals in this cohort. On T1 Gd, histogram features may reflect the degree and burden of contrast enhancement. This can be linked to vascular permeability and blood brain barrier disruption. On T2, similar features may capture the extent and heterogeneity of hyperintense components. This may relate to edema, infiltration, or necrotic and cystic changes. Nevertheless, these interpretations are indirect. They should be considered hypothesis generating. Future studies that integrate radiomics with molecular markers and pathology correlations will be important to refine the biological meaning of these imaging signatures.

Despite the promising findings, this study has several limitations that must be acknowledged. First, the analysis was conducted on a retrospective dataset of limited size from a single public archive. As we noted, the performance gap between our training and validation cohorts suggests a risk of model overfitting, which is a common challenge when working with small and potentially heterogeneous datasets. While we used repeated sub-sampling to mitigate this, the generalizability of our findings must be confirmed through external validation on a larger, prospective, multi-center cohort. A further limitation relates to the use of external segmentations and pre computed features. We did not independently evaluate segmentation accuracy. We also could not quantify inter rater variability within this study. Any systematic bias in the original segmentation strategy may influence the absolute performance of our models. This should be considered when interpreting the reported AUC values. At the same time, our main objective was a controlled comparison of sequence specific and fusion based feature sets. All models in our framework relied on the same upstream segmentations. This consistency helps reduce confounding in the relative comparisons across sequences. Future studies should assess segmentation sensitivity more directly. Further, we also acknowledge the use of a binary one-year survival endpoint. The main aim of this study was to examine the prognostic contribution of different MRI sequences under a controlled framework. It was not to build the best possible survival model. This simplified endpoint allowed fair comparisons across sequence combinations with fixed feature numbers and the same classifier. Nevertheless, time to event models may provide richer prognostic information and should be evaluated in future work.

In terms of clinical translation, the proposed dual sequence strategy using T1 Gd and T2 may offer a practical pathway for radiomics based survival stratification in GBM. Both sequences are routinely included in standard preoperative MRI protocols. This reduces barriers to implementation. It also avoids the need for additional scanning. If validated prospectively, this approach could be integrated into preoperative planning. It could serve as a decision support component. The model could provide an interpretable risk category. This may inform multidisciplinary discussions. It may also support patient counseling and trial stratification. Our findings suggest that a targeted two sequence fusion can achieve comparable generalization performance relative to full four sequence fusion in the validation setting. The performance may be slightly improved. This observation supports clinically streamlined and purpose-oriented sequence selection for specific predictive tasks. Future studies should prioritize prospective and ideally multicenter evaluation. These efforts should focus on workflow standardization and real-world feasibility. They should also assess robustness and interpretability in routine preoperative decision making. Nevertheless, we also emphasize that the present study is retrospective. It is based on publicly available multicenter data. Prospective validation will be essential before clinical deployment. This validation should use harmonized acquisition, segmentation, and feature harmonization pipelines. Future work will focus on establishing a standardized prospective workflow. We will also evaluate performance across centers in real world settings.

The findings from this study have several important implications for future research and potential clinical applications. Our results, which highlight the superior prognostic performance of a T1-Gd and T2 feature set, suggest that it may be possible to streamline MRI acquisition protocols for radiomic analyses in glioblastoma. If validated, a more focused imaging protocol could reduce patient scan times, lower costs, and improve the efficiency of data collection for clinical trials without sacrificing prognostic power. Furthermore, it is crucial to recognize that our conclusions are specific to this particular dataset and clinical objective. The applicability of these findings to other tumor types or different clinical tasks remains uncertain. Therefore, future research should incorporate more diverse datasets to investigate whether the superiority of the T1-Gd and T2 combination holds true across a broader range of oncological applications.

Our conclusions are based on glioblastoma and conventional structural MRI. The observed ranking of sequences and fusion strategies may not generalize directly to other glioma subtypes. Direct extrapolation may be insufficiently rigorous without dedicated validation. Lower grade gliomas differ in enhancement, infiltration, and molecular context. These factors may alter which sequences are most informative. Even so, our findings support a purpose oriented and streamlined approach to sequence selection. They suggest that adding more sequences is not automatically beneficial. Diffusion and perfusion imaging may provide complementary microstructural and hemodynamic information. Future work should test standardized integration of these modalities with structural radiomics across glioma subtypes.

## Conclusions

In this study, we systematically investigated the independent and combined prognostic value of radiomic features from four standard MRI sequences for survival classification in glioblastoma. Our results demonstrated that while the T2-weighted sequence provided the most performant single-sequence model, a dual-sequence fusion of features from T1-Gd and T2 images yielded the highest predictive accuracy overall. Notably, this targeted two-sequence combination outperformed the more complex model that aggregated features from all four sequences. In conclusion, our findings suggest that for glioblastoma survival prediction, the strategic selection of complementary imaging sequences, specifically the combination of T1-Gd and T2, is more effective than an indiscriminate aggregation of all available MRI data.

## Data Availability

The original contributions presented in the study are included in the article/supplementary material. Further inquiries can be directed to the corresponding author.
